# Macro- and micro-structural cerebellar and cortical characteristics of cognitive empathy towards fictional characters in healthy individuals

**DOI:** 10.1038/s41598-021-87861-0

**Published:** 2021-04-22

**Authors:** Eleonora Picerni, Daniela Laricchiuta, Fabrizio Piras, Daniela Vecchio, Laura Petrosini, Debora Cutuli, Gianfranco Spalletta

**Affiliations:** 1 IRCCS Fondazione Santa Lucia, Via Ardeatina 306, 00179 Rome, Italy; 2 Department of Psychology, Sapienza University, Via dei Marsi 78, 00185 Rome, Italy; 3 Division of Neuropsychiatry, Menninger Department of Psychiatry and Behavioral Sciences, Baylor College of Medicine, Houston, TX 77030 USA

**Keywords:** Neuroscience, Social neuroscience, Empathy

## Abstract

Few investigations have analyzed the neuroanatomical substrate of empathic capacities in healthy subjects, and most of them have neglected the potential involvement of cerebellar structures. The main aim of the present study was to investigate the associations between bilateral cerebellar macro- and micro-structural measures and levels of cognitive and affective trait empathy (measured by Interpersonal Reactivity Index, IRI) in a sample of 70 healthy subjects of both sexes. We also estimated morphometric variations of cerebral Gray Matter structures, to ascertain whether the potential empathy-related peculiarities in cerebellar areas were accompanied by structural differences in other cerebral regions. At macro-structural level, the volumetric differences were analyzed by Voxel-Based Morphometry (VBM)- and Region of Interest (ROI)-based approaches, and at a micro-structural level, we analyzed Diffusion Tensor Imaging (DTI) data, focusing in particular on Mean Diffusivity and Fractional Anisotropy. Fantasy IRI-subscale was found to be positively associated with volumes in right cerebellar Crus 2 and pars triangularis of inferior frontal gyrus. The here described morphological variations of cerebellar Crus 2 and pars triangularis allow to extend the traditional cortico-centric view of cognitive empathy to the cerebellar regions and indicate that in empathizing with fictional characters the cerebellar and frontal areas are co-recruited.

## Introduction

“*Every reader, as he reads, is actually the reader of himself*”

(Marcel Proust)

The non-motor role of the cerebellum has gained increasingly convincing evidence in the neuroscientific research. In fact, converging lines of experimental, clinical, and neuroimaging findings have advanced a new conceptualization on cerebellar functionality, indicating that the cerebellum is critically involved in a wide variety of cognitive, emotional and affective functions^1^, and also in social cognition. Indeed, neuroscientific evidence revealed a marked activation of the cerebellum during social judgments^2–10^ and functional connectivity studies evidenced strong neural connectivity between cerebellum and cerebrum during social interactions^11^ and social inferences^5,12–14^ as well as in the default/mentalizing network^2,3^.

Social cognition is a general term to describe cognition involving others, as understanding others’ emotions, intentions and behaviors and acting towards and with them in social settings^4,15^. Social cognition involves understanding others and understanding with others^15^. One of the most advanced social functions is the empathic capacity that allows sharing the affective states of others, exerting cognitive control, predicting, and understanding others’ feelings, motivations and actions, and behaving accordingly^16^. In humans and non-human animals, the empathy is aimed at promoting prosocial and cooperative behaviors^16–19^. This psychological construct is regulated by both affective and cognitive components that produce the emotional understanding^20^. Affective empathy refers to the ability of sharing the state of other persons through observation or imagination of their experience. Such ability usually leads to an appropriate isomorphic emotional response as a consequence of other's state. Cognitive empathy refers to the abilities of perspective taking and Theory of Mind (ToM) that allow predicting and understanding other's mental state by using cognitive processes. Combined, these processes enable to understand beliefs, desires, and emotions of others in real-life or imaginary situations, although the subject remains aware that someone else is the source of that state.

Evidence from functional Magnetic Resonance Imaging (fMRI) studies^21^ and functional connectivity measures^22–24^ have indicated consistent activation of specific brain structures (frontal, parietal and temporal neocortical structures) associated with each component of empathy. Namely, the anterior cingulate cortex (ACC) and anterior insula (AI) are mostly recruited in the affective empathy, whereas the medial cingulate cortex and adjacent dorsomedial prefrontal cortex (MCC/DMPFC) in the cognitive empathy. While many studies have assessed empathy as a state rather than a trait focusing on the neuronal activation associated with empathy-eliciting situations^25–27^, few investigations have been interested in the structural underpinning of the trait empathy, and additionally most often in the presence of pathological conditions, as schizophrenia^28^, aggressive and antisocial behavior^29^, neurodegenerative disorders^30^. To date, very few reports have analyzed the brain substrate of empathic capacity in healthy subjects by using Voxel-Based Morphometry (VBM)^31–34^. It is evident that the research on the neural correlates of empathy has been so far mainly focused on the cerebrum, however the increasingly recognized involvement of cerebellum in social cognition^1,6,12^, of which empathy is one of the most advanced component, makes it necessary aimed investigations on the role played by the cerebellum in empathic capabilities. In support of this idea, empathy for other people’s feelings of pain is associated with cerebellar activation in fMRI studies^35–38^, and bilateral lesions to posterior vermis and cerebellar hemispheres result in empathy and ToM deficits^39,40^. Although it is widely accepted that the cerebellum is implicated in many neuropsychiatric disorders featured by social malfunctioning, such as autistic spectrum disorders, attentional deficit and hyperkinetic disorder, depression, and schizophrenia^1,41,42^, only recently it has been recognized that the cerebellum plays a more critical role in social thinking than assumed so far. In meta-analytic studies on fMRI studies in healthy humans on cerebellum and social cognition, a robust activation of the cerebellar areas during social judgments and mentalizing has been identified^4,5,8,8,14^. One may wonder whether social processes activate the cerebellum in a distributed fashion at many locations or they activate a specialized cerebellar function and recruit limited areas. Recent evidence has documented that as specific areas of the cerebellum are specialized in motor processing and others in cognitive and affective functions, there are areas in the posterior cerebellum (Crus 1 and 2) selectively recruited during social cognition. Interestingly, in these cerebellar areas a distinct mentalizing network, directly connected to the cerebral mentalizing network, has been identified^3^.

In the present research we investigated whether and which cerebellar regions are involved in the empathic capabilities by analyzing the associations between cerebellar macro- and micro-structural measures and levels of affective and cognitive trait empathy (measured by Interpersonal Reactivity Index, IRI)^17^ in a sample of 70 healthy subjects of both sexes. We also estimated the eventual empathy-related morphometric modifications in extra-cerebellar structures. While at macro-structural level the volumetric differences were analyzed through both whole brain- and Region Of Interest (ROI)-based analyses, at a micro-structural level Diffusion Tensor Imaging (DTI) was analyzed through a ROI-based approach. DTI supplies reliable physiological information on the direction and degree of water displacement in the brain, providing thus information on the obstacles encountered by diffusing water molecules^43,44^. Among DTI indices, Mean Diffusivity (MD) and Fractional Anisotropy (FA) were used as probes for, respectively, Gray Matter (GM) and White Matter (WM) micro-structural integrity^45–48^. In this perspective, DTI measures represent a reliable research tool that supplies physiological information not available on conventional MRI.

## Results

### Sociodemographic and Psychological Variables

Mean scores and standard deviations of each psychological variable (IRI subscales, HAM-A and HAM-D) are reported in Table 1. While gender differences on HAM-A (*t* = 1.11; p = 0.268) and HAM-D (*t* = 1.20; p = 0.231) data were not significant, those on each IRI subscale indicated that females showed significantly higher scores than males in Fantasy, Personal Distress, and Empathic Concern (Tables 1 and 2).

**Table 1:** Scores on psychological instruments (IRI subscales, HAM-A and HAM-D) for all subjects, males and females (mean ± standard deviation).

IRI subscale	All participants	Males	Females
Fantasy	14.44 ± 5.20	12.80 ± 5.45	15.74 ± 4.66
Perspective taking	18.28 ± 3.08	17.58 ± 3.08	18.84 ± 3.00
Empathic concern	18.08 ± 2.98	17.19 ± 3.00	18.79 ± 2.80
Personal distress	9.68 ± 4.64	8.09 ± 3.57	10.94 ± 5.04
HAM-A	4.82 ± 3.81	4.25 ± 3.94	5.28 ± 3.69
HAM-D	2.78 ± 2.66	2.35 ± 2.78	3.12 ± 2.55

**Table 2:** IRI subscales and sociodemographic variables.

IRI Subscales	Age		Years of education		Gender		HAM-A		HAM-D	
	*r*	*p*	*r*	*p*	**t**	*p*	*r*	*p*	*r*	*p*
Fantasy	− 0.03	0.816	0.10	0.400	− **2.38**	**0.020**	0.17	0.155	0.03	0.813
Perspective taking	**− 0.29**	**0.015**	**0.30**	**0.010**	− 1.72	0.089	− 0.18	0.119	− 0.17	0.159
Empathic concern	0.19	0.105	− 0.00	0.945	− **2.28**	**0.026**	− 0.03	0.826	− 0.03	0.803
Personal distress	0.02	0.864	− 0.04	0.736	− **2.76**	**0.007**	**0.27**	**0.023**	**0.34**	**0.003**

Significant results (p < 0.05) are in Bold.

As expected, significant direct correlations were found between some IRI subscales. Namely, the scores of Perspective Taking positively correlated with Empathic Concern (r = 0.24; p = 0.047); Fantasy positively correlated with Empathic Concern (r = 0.41; p < 0.001) and Personal Distress (r = 0.41; p < 0.001). Also, significant correlations were found between scores of the IRI subscale Perspective Taking and age and education levels. Personal Distress showed a significant direct association with HAM-A and HAM-D scores (Table 2).

### ROI-based VBM

**Figure 1:**
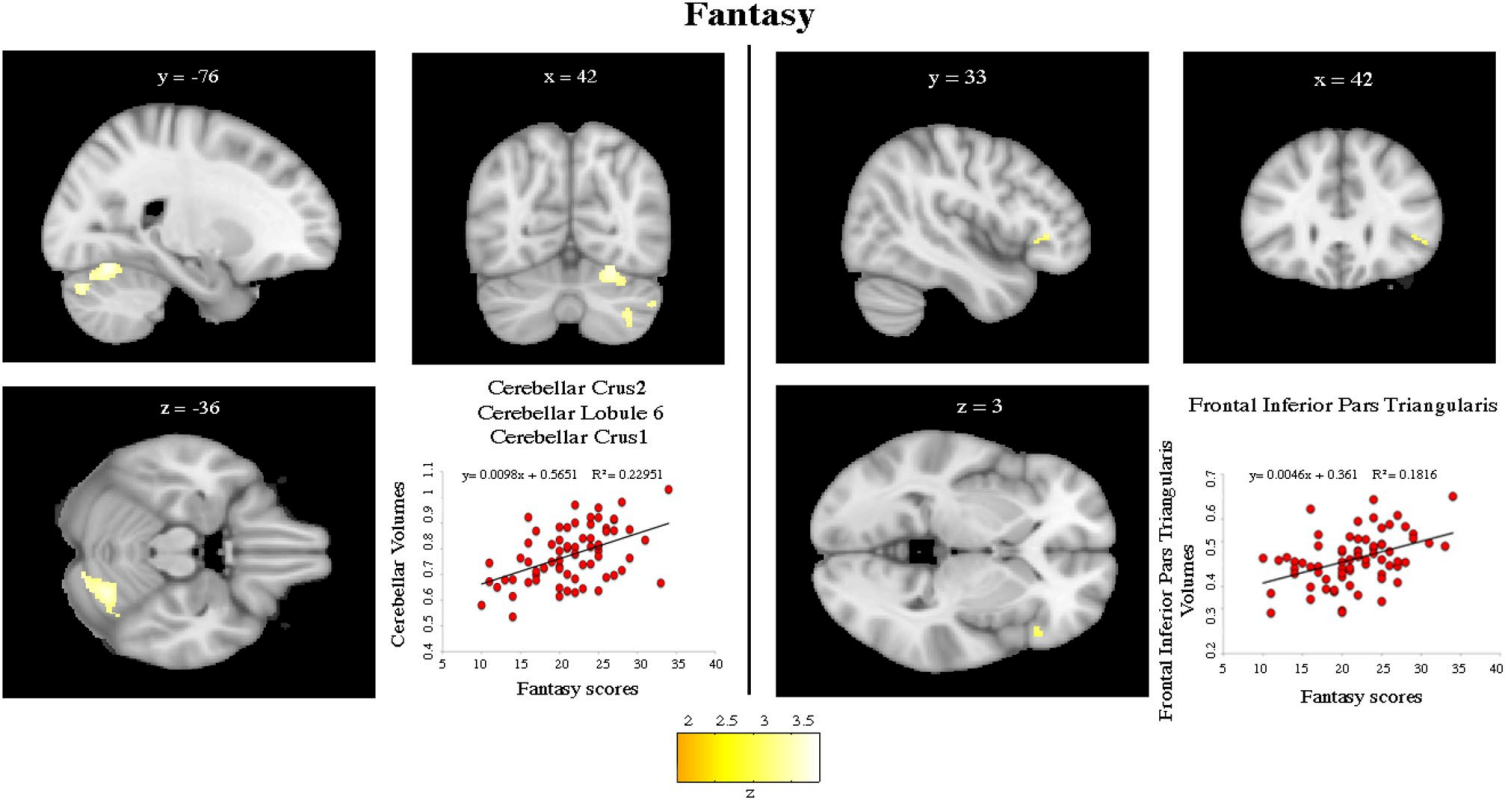
Positive association between a priori Regions of Interest (ROIs) and Fantasy IRI subscale. Coordinates are in Montreal Neurological Institute (MNI) space. Z below colorbar indicates normalized t-values. In figure left is left. Areas significantly associated with Fantasy subscale in the ROI-based analyses were used as masks to extract raw data and create scatterplot. Equation, R^2^ and linear fit (solid black line) are reported.

Analyses on the a priori selected areas revealed significant associations. Namely, positive associations were found between Fantasy IRI subscale and an extended (1271 voxels) cerebellar cluster in right Crus 2, Crus 1, and Lobule 6 (pFWE_corr_ = 0.009) (Fig. 1; Table 3). Furthermore, Fantasy IRI subscale was positively correlated with right frontal inferior pars triangularis (pFWE_corr_ = 0.029) (Fig. 1; Table 3).

**Table 3:** Regional gray matter volumes (ROI-based and voxel based morphometry analyses) and fantasy IRI subscale.

Label for peak	Side	Extent (n voxels)	t	*p*	equivZ	x,y,z (mm)
**ROI-based**						
Cerebellum Crus 2 (Crus1, Lobule 6) ↑	R	1271	3.88	**0.009**	3.67	42, − 76, − 36
Inferior frontal gyrus, Pars Triangularis ↑	R	54	4.24	**0.029**	3.97	42, 33, 3
**Voxel based morphometry**						
Cerebellum Crus 2 ↑	R	1709	3.93	**0.002**	3.71	44, − 76, − 38

Results are FWE corrected.

Coordinates are in Montreal Neurological Institute (MNI) space.

*L* left, *R* right.

*p* = significance (FWE corrected) at the cluster level (Cerebellum Crus 2) and at the peak level (Inferior Frontal Gyrus, Pars Triangularis).

Significant results (p < 0.05) are in Bold.

The associations significant at uncorrected statistical level between IRI subscales and ROI-based VBM data are reported as Supplementary Materials S1.

### Whole-brain VBM

A positive association was found between Fantasy IRI subscale and an extended (1709 voxels) cerebellar cluster in right Crus 2 (pFWE_corr_ = 0.002) (Table 3).

The associations significant at uncorrected statistical level between IRI subscales and whole-brain VBM data are reported as Supplementary Materials S1.

### DTI Analyses

**Figure 2:**
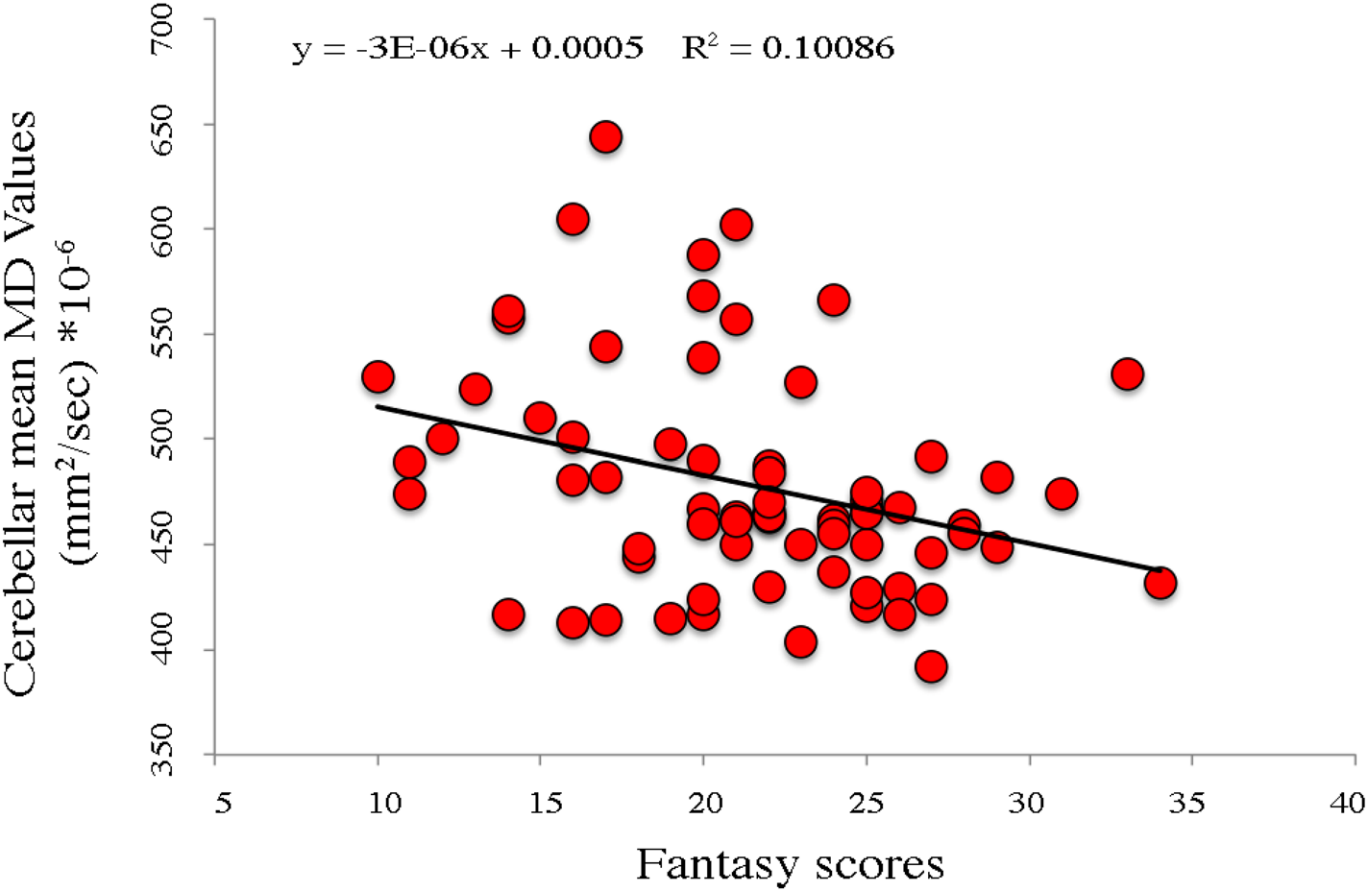
Negative association between cerebellar Crus 2 mean Mean Diffusivity (MD) values and Fantasy IRI subscale scores. Equation, R^2^ and linear fit (solid black line) are reported.

MD values of right cerebellar Crus 2 showed a significant negative association with Fantasy subscale scores (r = 0.30; p = 0.010) (Fig. 2). Such an association remained significant even when controlled for age and gender (R^2^ = 0.181; p = 0.028). FA values failed to reveal any significant association with cerebellar areas. No significant associations were found between Fantasy scores and both MD and FA micro-structural measures of right pars triangularis.

## Discussion

The main result of the present research is that the volumes in right cerebellar Crus 2 and pars triangularis of inferior frontal gyrus were positively associated with the Fantasy IRI-subscale scores. The increased volumes in Crus 2 were accompanied by diminished MD values. An MD decrease is generally considered to reflect increased functional capacities. In fact, lower MD values across the whole brain are associated with higher IQ scores, and lower MD values in the motivation-related subcortical areas (putamen and pallidum) are associated with greater motivational state^49^. Conversely, higher MD values in basal region of putamen are associated to increased anxiety-related personality traits^46,50^. Thus, a pattern of increased volume and diminished MD such as the one we found in Crus 2 speaks in favor of a greater functional capacity.

Since the trait empathy is expressed with variable intensity in the healthy population, we expected an effect size of cognitive and affective empathy on brain morphology to be small. Conversely, the associations of Fantasy subscale scores with cerebellar and frontal (pars triangularis) volumes were powerfully significant. Taking into account the close anatomical and functional proximity of the pars triangularis (BA45) and dorsolateral prefrontal cortex (DLPFC) (BA46), it appears notable the positive association between right DLPFC volume and Fantasy subscale scores^31^ and the role of right DLPFC in identification process measured with the same subscale^32^. Unfortunately, both studies have neglected any possible association between Fantasy subscale and cerebellar volumes.

Although healthy, all participants were also evaluated by HAM-D and HAM-A scales^51,52^. A positive correlation was found only between scores of Personal Distress and both HAM scales, in line with previous reports^53–55^. Thus, the more the self-oriented perspective traits were present, the more the anxious and depressive tendencies were evident. This finding suggests that in the presence of others’ suffering the self-orientation may induce ruminations on what it would be like to be affected oneself, resulting in the feeling of negative affect and distress^56,57^.

Interestingly, a recent fMRI study reports the activation of right Crus 1 and 2 when the participants viewed a short dramatic movie rich in dynamic cognitive and affective contents^58^. The activity in right Crus regions correlated with the presence of a structured storyline and with distinct unexpected features of the movie, such as turning points of the plot and sequences with verbal components. Convincingly, the authors have interpreted their results as an index of the cerebellar engagement in dynamic perceptual and affective processes elicited by naturalistic stimuli, such as movies, spoken or read narratives. In accordance with this evidence, the increased volumes in Crus 2 associated with high scores of Fantasy subscale may represent the structural underpinning of the processes required for empathizing with characters of immersive storylines.

Reading a book or watching a movie are non-innate and highly artificial brain activities, which may occupy a very significant part in the daily life of many people, presumably because their considerable adaptive value^59^. During such activities it is often opened a window into characters’ thoughts and feeling, so that people respond in though and feeling to fictive situations, as if they actually occur. Ultimately, these activities help subjects to optimize decisions and actions, learn about existing or fictive worlds, and stimulate motivation and imagination, functioning thus as a sort of “emotional gym”. In watching a movie or reading a book subjects may be so emotionally moved to get lost in the fictive happenings of the stories as if these were real, and imaginatively perceive themselves as transposed into character’s thoughts and feelings, experiencing the character’s happenings from the character’s perspective, and merging with or being that character. The cognitive empathy required for such processes is measured by the IRI subscale Fantasy.

The cerebellar involvement in cognitive empathy fits with the significant covariation of right lateral cerebellum with self-rated individual differences in empathy for pain described by Singer and colleagues^38^, and with the reported cerebellar involvement in social cognition^4,7,60^. Even the new functional parcellation of the cerebellar cortex proposed by King and colleagues^61^ evidenced the activation throughout right Crus 1 and 2 with ToM tasks that are essential in social interactions. Correspondingly, bilateral lesions of cerebellar posterior vermis and hemispheres result in empathy and ToM deficits^40^. In a pediatric brain injury sample the individual differences in cerebellar volumes predicted ToM outcomes, and the volumetric reductions in Cerebro-Cerebellar Mentalizing Network (CCMN) predicted poor ToM performances^62^. Some neuropsychiatric disorders exhibiting cerebellar dysfunctions display impairments in social functions. For instance, the performance of patients with various types of cerebellar damage is impaired in ToM tasks^63^. In children affected by autism spectrum disorders VBM analyses revealed reduced GM volumes in right Crus 1 and 2 and emphasized that the degree of cerebellar GM reductions correlated with the severity of symptoms in social interactions, communication, and repetitive behaviors^41^. More specifically, it has been recently reported that the size of empathic imbalance between the cognitive and emotional components is positively correlated with autism traits in neurotypical population^64^. Furthermore, cognitive empathy predominant on affective empathy is related to stronger connectivity in interoceptive and socio-cognitive networks including the cerebellum^65^. Additionally, in a fMRI study individuals with alexithymia (i.e. difficulty in recognizing and expressing one’s own emotions and in describing the emotional experience of others in hypothetical situations^66^) showed decreased activation in the cerebellum (and increased activation in the anterior insula) in response to empathic painful situations in comparison to healthy controls^37^. On the whole, these data highlight the involvement of the cerebellum in socio-cognitive processes^6,14,67,68^ and support the view of a “social cerebellum”, and more specifically of an “empathic cerebellum”.

In motor domain, the cerebellar networks construct internal models of motor processes to control whether actions are executed as planned; if it is not, the generation of an error signal regulates cortical processes responsible for that error, facilitating thus the enacting of the intended action. Notably, the cerebellum forms forward or inverse internal models even of mental processes without involvement of overt movements and somatosensory responses^69^. The internal models are neural representations that encode the context-specific dynamics of concrete or abstract representations to facilitate predictive control of the system. The cerebellar-dependent, error-based learning is used to calibrate forward models. Therefore, fundamental cerebellar functions are predicting the consequences and scope of motor and non-motor operations by reconstructing hypothetical events, and signaling prediction errors to the cortex. To successfully manage any mismatch, the co-activation of cerebellum and neocortical areas appears needed since the predictions are based on information from the cortex to the cerebellum (efferent copies), and error signals are sent from the cerebellum to the cortex. Similar to what happens in the sensorimotor system, it has been advanced that the related processes of forward modelling and error sensitivity may characterize the cerebellar function even within social realm allowing thus to anticipate the other’s behavior or one’s own reactions^8^^,^^63,70,71^*.* On the basis of the present findings we are advancing that these concepts of prediction and error processing may be advanced to understand the cerebellar contribution even to empathy with fictional characters. When the subject empathizes with fictional characters the cerebellar forward model potentially generates representations and predictions regarding the feelings of the character. Internal models develop by using past perceptual, motor, and socio-emotional experiences of the empathizer framed by the intentions, beliefs, and feelings of the character. The degree of matching between subject and character relies on such representations, but the subject can efficiently match with the state of the character to the degree that s/he has already existing representations for that state, pointing out the experience-dependence of such a process, in analogy to what previously described for the motor domain^72^. In their fMRI study, Calvo-Merino and colleagues have examined the brain activity of expert dancers who watched videos of ballet moves and have observed greater activity in right cerebellum and premotor areas when subjects were looking at the movements they were more familiar with, indicating that observing others’ actions engages neural pathways associated with sensorimotor internal models. Ultimately, the internal models involving cortico-cerebellar networks may also be essential for empathic responses in real-life or imaginary situations.

Notably, in addition to volumes of right Crus 2 we found that Fantasy subscale scores were powerfully associated with volumes of right pars triangularis (BA45) of the inferior frontal gyrus (BA44, BA47). As known, the functions of these frontal areas are related to the ability to live sociably and communicate with others, being key nodes of the mirror neuron system (MNS)^73–75^. Given its observation-execution matching properties, MNS provides the appropriate mechanism for empathy and imitation^76^, and allows identifying goals and intentions of others by their resemblance to stored representations for the same states (experience-dependence). MNS may facilitate thus the simulation of behavior—even social—of the other^77–80^, even when the other is a fictional character. Reading about or viewing a character who experiences a powerful emotion stimulates mirroring mechanisms and through the implementation of the internal models (provided by the cerebellum) might form embodied representations of that emotion grounded in perceptual, sensorimotor, and visceral control loops^81^. These embodiment circuitries act as a boost for subsequent socio-emotional processes, allowing the remapping of character's states into the corresponding subject’s sensorimotor and visceral brain areas, making the subject experience the same emotion of the fictional character^18,82–84^. The more similar the character’s state is to something the subject has already experienced, the more his/her representations will match character’s state^18^. Consistently with these considerations, it has been postulated that the prefrontal areas activate when two or more emotional states—such as one's own and that of the other (in our case the fictional character) are simultaneously processed and integrated to form a higher-order empathic state^20^. It has been described the engagement of the right inferior frontal cortex when comparing conditions in which the subject attributes a mental state to a character in a story in which s/he is featured and one in which s/he is absent^85^. The critical role of the right inferior frontal cortex in the inhibition of self-perspective has been also described, reporting a case of a subject with a lesion of this area who was impaired in ToM tasks that required the suppression of his own perspective but performed well if they did not^86^.

Empathy toward fictional characters slightly differs from empathy towards real people due to the need in the first case to overcome the difficulties in attending to internal over external stimuli and to bring character’s state into mind to succeed in feeling it. In this sense, since movies and stories offer narratives that are unfolding in time, empathizing with a fictional character requires the maintenance (or the internal rehearsal) of information in working memory, once again engaging the prefrontal networks, including the pars triangularis^87^. Anyway, working memory system functions only when it is driven by attention. It has been shown that top-down attention to one’s own emotions activates the prefrontal cortical areas^87–90^. Then, the notion that the more attentive the subjects are, the more they are drawn into the character’s emotional situation emphasizes the importance of the cerebello-frontal processing in the proper attentional maintenance (or shift) required for empathic response to occur. In fact, the cerebellum (and its connections with prefrontal areas) is necessary to execute attentional shifts, to track the state of the character, and to avoid attending to misdirection and intruders, to stay focused on the plot.

A final consideration has to be added. In watching a movie, or listening to or reading a story, it is necessary to accumulate, integrate and process a lot of information (often with high socio-emotional content) in order to gain cognitive and emotional comprehension of that story^91–93^. The high-order prefrontal, temporal and parietal areas activated in these situations coincide with the members of the DMN, the network that regulates the switch from an internal reference state to external target-oriented behaviors. Remarkably, even the cerebellum, and in particular Crus 2, belongs to this network as indicated by its coherent activation with the cortical areas of DMN^3,5^. Connectivity studies revealed that function-specific cerebellar networks are strongly connected to cortical networks serving the identical function^5^. Since DMN involves cortical and cerebellar activity, the volumetric increased volume in Crus 2 and frontal pars triangularis associated with the enhanced empathizing abilities with fictional characters found in the present research appears intriguing.

## Conclusions

Emotionally charged narratives or movies allow re-experiencing emotions in the resonating sensory-motor systems of the subject, as if s/he were there in the same situation, experiencing the very same emotional state of the other, even if the other is a fictional character. The present findings indicate that the empathy with emotionally charged characters is enabled by the coordinated activity of cerebellar areas (Crus 2) and MNS (pars triangularis). These findings are in line with the “fiction feeling hypothesis”^59^ that posits that greater emotionality in a narrative results in greater feelings of empathy and immersion and in greater recruitment of networks of cognitive mentalizing empathy^94^.

## Methods

### Participants

A sample of 70 healthy subjects (31 males; mean age ± SD: 41.11 ± 12.34 years; range: 21–62) was recruited for the study. Educational level ranged from an eighth grade to a post-graduate degree (mean education years ± SD: 15.83 ± 2.86; range: 8–25). All participants were right-handed as assessed with the Edinburgh Handedness Inventory^95^. Inclusion criteria were: age between 18 and 65 years and suitability for MRI scanning. Exclusion criteria included: (1) cognitive impairment or dementia, based on Mini Mental State Examination (MMSE)^96^ scores ≤ 24^97^, and confirmed by clinical neuropsychological evaluation by using the Mental Deterioration Battery^98^ and the NINCDS-ADRDA criteria for dementia^99^; (2) subjective complaint of memory difficulties or of any other cognitive deficit, regardless of interference with daily activities; (3) major medical illnesses, e.g. diabetes (not stabilized), obstructive pulmonary disease, or asthma; hematologic and oncologic disorders; pernicious anemia; clinically significant gastrointestinal, renal, hepatic, endocrine, or cardio-vascular system diseases; newly treated hypothyroidism; (4) current or reported mental (assessed by SCID-I and the SCID-II)^100^ or neurological (assessed by clinical neurological evaluation) disorders (e.g. schizophrenia, mood disorders, anxiety disorders, stroke, Parkinson’s disease, seizure disorder, head injury with loss of consciousness, and any other significant mental or neurological disorder); (5) known or suspected history of alcoholism or drug dependence and abuse, evaluated by structured interviews (SCID I or SCID II)^100,101^; (6) MRI evidence of focal parenchymal abnormalities or cerebro-vascular diseases: for each subject, a trained neuroradiologist and a neuropsychologist expert in neuroimaging co-inspected all the available clinical MRI sequences (i.e. T1- and T2-weighted and FLAIR images) to ensure that the subjects were free from structural brain pathologies and vascular lesions (i.e. FLAIR or T2-weighted hyper-intensities and T1-weighted hypo-intensities).

### Ethical statement

In accordance with the Declaration of Helsinki the study was approved by the Local Ethics Committee of Santa Lucia Foundation IRCCS. We confirm that the whole research was performed in accordance with relevant guidelines/regulations and that written consent was obtained from all participants after full explanation of study procedures.

### Psychological Instruments

#### Trait empathy assessment

Empathic abilities were assessed through the Interpersonal Reactivity Index, IRI^17^, a widely used well-validated, multidimensional measure of trait empathy. The questionnaire is based on a self-report comprising 28 items answered on a 5-point Likert scale ranging from 0 ("Does not describe me well") to 4 ("Describes me very well"). The measure has 4 subscales, each made up of 7 different items, and for each subscale, a minimum score of 0 or maximum score of 28 is possible. The subscales that measure the affective dimension of empathy are *Personal Distress* and *Empathic Concern,* while the subscales that measure the cognitive dimension of empathy are *Perspective Taking* and *Fantasy*. In more detail, Personal Distress is “self-oriented” and is associated to aversive emotional responses in the observer (e.g. feelings of fear or discomfort at witnessing negative experiences of others) (Sample item: *When I see someone who badly needs help in an emergency, I go to pieces*), while Empathic Concern is “other-oriented” and is related to feelings of compassion and sympathy for observed unfortunate individuals *(I often have tender, concerned feelings for people less fortunate than me*). Perspective Taking examines the tendency to spontaneously adopt the psychological point of view of others in everyday life (i.e. cognitive responses) (*Before criticizing somebody, I try to imagine how I would feel if I were in their place)*, while Fantasy examines participants' abilities to imaginatively transpose themselves into feelings and actions of fictitious characters in books, movies, and plays *(When I am reading an interesting story or novel, I imagine how I would feel if the events in the story were happening to me*).

#### Depression and anxiety assessment

Because of the known associations between empathy and depression/anxiety^55,102–104^, although healthy, all participants were evaluated by means of Hamilton rating scales. Namely, presence and severity of depressive symptoms were evaluated by using Hamilton depression rating scale‐17 items (HAM‐D17, indicated in the text as HAM-D). Scores < 8 indicated no depression, scores from 8 to 17 corresponded to mild depression, scores from 18 to 24 corresponded to moderate depression, and scores > 24 severe depression^51^**.** Presence and severity of anxiety symptoms were evaluated by using Hamilton anxiety rating scale (HAM-A), which consists of 14 questions. Scores < 5 indicated no anxiety, scores between 6 and 14 indicated mild anxiety, and score > 14 indicated moderate to severe anxiety^52^.

### Image acquisition

All participants underwent the imaging protocol originally described elsewhere^45,47,48^. The protocol included standard clinical sequences (FLAIR, DP-T2-weighted), a volumetric whole-brain 3D high-resolution T1-weighted sequence, and a DTI scan protocol, performed with a 3-T Achieva MR imager (Siemens, Erlangen, Germany). Volumetric whole-brain T1-weighted images were obtained in the sagittal plane using a modified driven equilibrium Fourier transform (MDEFT) sequence (Echo Time/Repetition Time—TE/TR— = 2.4/7.92 ms, flip angle 15°, voxel size 1 × 1 × 1 mm^3^). Diffusion volumes were acquired by using echo-planar imaging (TE/TR = 89/8500 ms, bandwidth = 2126 Hz/vx; matrix size 128 × 128; 80 axial slices, voxel size 1.8 × 1.8 × 1.8 mm^3^) with 30 isotropically distributed orientations for the diffusion-sensitizing gradients at one b value of 1000 s mm^2^ and two b = 0 images. Scanning was repeated three times to increase the signal-to-noise ratio. All planar sequence acquisitions were obtained in the plane of the anterior–posterior commissure line. Since the posterior cranial fossa usually falls at the lower limit of the field of view, particular care was taken to center subjects’ head in the head coil, in order to avoid possible magnetic field dishomogeneities or artifacts at the level of the cerebellum.

### Image processing

T1-weighted and DTI images were submitted to several processing steps. First, to explore the relationship between regional volumes and empathy on a voxel-by-voxel basis, T1-weighted images were processed and examined using the SPM8 software (Wellcome Department of Imaging Neuroscience Group, London, UK; http://www.fil.ion.ucl.ac.uk/spm), specifically the VBM8 toolbox (http://dbm.neuro.uni-jena.de/vbm.html) running in Matlab 2007b (MathWorks, Natick, MA, USA). The toolbox extends the unified segmentation model^105^ consisting of MRI field intensity inhomogeneity correction, spatial normalization, and tissue segmentation at several pre-processing steps to further improve data quality. Initially, to increase the signal-to-noise ratio, an optimized block-wise nonlocal-means filter was applied to the MRI scans using the Rician noise adaption^106^. Then, an adaptive maximum a posteriori segmentation approach extended by partial volume estimation was employed to separate the MRI scans into GM, WM, and cerebro-spinal fluid. The segmentation step was finished by applying a spatial constraint to the segmented tissue probability maps based on a hidden Markow Random Field model to remove isolated voxels, which unlikely were members of a certain tissue class, and to close holes in clusters of connected voxels of a certain class, resulting in a higher signal-to-noise ratio of the final tissue probability maps. Then, the iterative high- dimensional normalization approach provided by the Diffeomorphic Anatomical Registration through Exponentiated Lie Algebra (DARTEL)^107^ toolbox was applied to the segmented tissue maps to register them to the stereotaxic space of the Montreal Neurological Institute (MNI). The tissue deformations were used to modulate participants’ GM and WM maps to be entered in the analyses. Voxel values of the resulting normalized and modulated GM and WM segments indicated the probability (between 0 and 1) that a specific voxel belonged to the relative tissue. Finally, the modulated and normalized GM and WM segments were written with an isotropic voxel resolution of 1.5 mm^3^ and smoothed with a 6-mm Full-Width Half Maximum (FWHM) Gaussian kernel. The segmented, normalized, modulated and smoothed GM and WM images were used for analyses. Subsequently, DTI data were pre-processed and analyzed in Explore DTI v4.8.6^108^. Data were corrected for motion and eddy currents. Motion artifacts and eddy current distortions were corrected with B-matrix rotation using the approach by Leemans and Jones^108^. During this processing procedure, all brain scans were rigidly normalized to Montreal Neurological Institute (MNI) space during the motion-distortion correction step. A diffusion tensor model was fit at each voxel and maps of FA and MD were generated. All diffusional indexes were finally written in a resolution of 2 × 2 × 2 mm. MD and FA maps were subsequently smoothed by using a Gaussian kernel with a 6-mm FWHM. Among DTI indices, Mean Diffusivity (MD) and Fractional Anisotropy (FA) were used as probes for GM and WM micro-structural integrity, respectively^45–48^. MD measures the averaged diffusion of water molecules through tissues providing information on restrictions (e.g., high density of cells) that water molecules encounter. If these obstacles have coherent alignment, on average the water tends to diffuse more along a certain axis. MD reflects cellular and cyto-architectonic changes, which result in higher density of synapses, spines, and capillaries, modifications in the properties of myelin and membranes, alterations in shape of glial cells and neurons. Ultimately, decreased MD reflects increased functional adaptation, and increased MD has been linked to poor cognitive performance or psychiatric symptoms^109^ and to states characterized by reduced efficacy of synaptic and extra-synaptic transmission^110^. FA measures the anisotropy of water diffusion processes and it is positively linked to fiber density, axonal diameter and myelination in WM^43^. Low FA values stand for isotropic diffusion (i.e., unrestricted in all directions), while high FA values indicate diffusion fully restricted along one axis.

### Statistical analysis

#### Sociodemographic and psychological variables

Parametric associations between IRI scores and age, years of formal education, and HAM-D and HAM-A scores, were analyzed by Pearson’s product moment correlations (Fisher’s r to z). Gender Differences in psychological variables were assessed by unpaired t test. Results of the demographic characteristics were considered significant at the p < 0.05 level.

#### Volumetric analyses

##### ROI-based VBM

We selected ROIs considering previous functional and structural neuroimaging studies on empathic abilities to constrain our anatomical hypotheses. In particular, as main aim of the present study we focused our analyses on the cerebellum. As ancillary aim we analyzed the anterior cingulate cortex^38^, inferior frontal gyrus^26^, precuneus^111^, insula^38^, somatosensory cortex^112^, medial prefrontal cortex^26,113^, supplementary motor area^38^, and frontal inferior pars triangularis^114^. These regions were selected based on the quoted functional neuroimaging studies, demonstrating their involvement in affective and cognitive empathy, and meta-analyses^113^ of brain regions involved in affect sharing and mentalizing^31^.

The MNI-oriented atlas of the human brain (Automated Anatomical Labeling Atlas, AAL)^115^ was used to extract GM masks of the ROIs singularly achieved by meaning all GM probability maps, obtained in the VBM8 processing steps, thresholding the relative image to a value of 0.3 (i.e. removing all voxels having a probability to belong to GM lower or equal to 29%), and manually removing all the other structures (e.g. for the cerebellum by manually removing all the non-cerebellar structures) using the AAL template, as reference. The resulting data were then fed into VBM analyses to evaluate morphological changes associated with ROIs and empathy subscales. We evaluated at the voxel-level the associations between cerebellar or neocortical (either ROIs or whole-brain VBM) structural measures and empathy scores, by using SPM8 within the framework of the General Linear Model. Multiple-regression analyses were computed by singularly using the measures of ROIs GM volumes as dependent variables, the scores of empathy IRI subscales as regressors, and age as covariate to control age impact on the brain structures. Moreover, when significantly associated to empathy IRI subscales, also gender, education years, depression or anxiety levels were used as covariates. Gender was always considered a ‘‘dummy variable’’ given its dichotomic nature.

##### Whole-brain VBM

As for ROI-based VBM analyses, also whole-brain VBM multiple-regression analyses were computed by singularly using the measures of GM volumes as dependent variables, the scores of IRI subscales as regressors, and age as covariate. When significantly associated to empathy IRI subscales, also gender, education years, depression or anxiety levels were used as covariates. We considered significant only the relationships whose voxels were part of a spatially contiguous cluster size of a minimum of 50 voxels, and that survived (p < 0.05) at the Family Wise Error (FWE) correction. Anyway, to avoid the risk of type II errors, in Supplementary Materials S1 we reported the areas significantly associated at uncorrected statistical level (p_uncorr_ < 0.001) to scores of empathy IRI subscales.

To obtain the precise anatomical localization of VBM results, we superimposed statistical maps onto Diedrichsen’s probabilistic atlas of the human cerebellum, which subdivides the cerebellum into ten different regions^116^ or onto the AAL template for extra-cerebellar ROIs and whole-brain analyses. Finally, the mean values of GM volumes significantly associated (pFWE_corr_) with empathy scores in ROIs and whole-brain analyses were extracted and used to create scatterplots.

#### DTI analyses

The areas significantly associated (pFWE_corr_) with IRI subscales at macro-structural analyses were used as masks and applied to MD and FA maps, in order to extract mean micro-structural values for each measure. Parametric associations between empathy scores and mean MD or FA values were analyzed by Pearson’s product moment correlations (Fisher’s r to z) to assess potential significant associations also with micro-structural measures. Analyses were also controlled for age, and, when significantly associated to IRI scores, also for gender, education years, depression or anxiety levels, as above described. Then, MD or FA values significantly associated with IRI scores were extracted and used to create scatterplots.

## Supplementary Information

Supplementary Information
