# Supplementary material for: Macro- and micro-structural cerebellar and cortical characteristics of cognitive empathy towards fictional characters in healthy individuals
Source: Sci Rep. 2021 Apr 22;11:8804. doi: 10.1038/s41598-021-87861-0 (PMC8062506; doi:10.1038/s41598-021-87861-0)
Supplement: Supplementary file 1 — Supplementary Information [file 41598_2021_87861_MOESM1_ESM.docx]

**Macro- and micro-structural cerebellar and cortical characteristics of cognitive empathy towards fictional characters in healthy individuals**

**Eleonora Picerni^1^°, Daniela Laricchiuta^1^, Fabrizio Piras^1^, Daniela Vecchio^1^, Laura Petrosini^1^,**

**Debora Cutuli^1,2^* and Gianfranco Spalletta^1^***

**^1^ IRCCS Fondazione Santa Lucia, Via Ardeatina 306, 00179, Rome, Italy**

**^2^ Sapienza University, Via dei Marsi 78, 00185 Rome, Italy**

ROI-based VBM

At an uncorrected level of significance (p_uncorr_ < 0.001), Fantasy IRI subscale was positively associated with the left precuneus. Positive uncorrected associations (p_uncorr_ < 0.001) were also found between Personal Distress and two clusters in left and right somatosensory cortex, and between Empathic Concern and left somatosensory cortex. Negative uncorrected associations (p_uncorr_ < 0.001) were found between Perspective Taking and two clusters in right supplementary motor area and one in left somatosensory cortex (Supplementary Table 1).

Whole-brain VBM

At an uncorrected level of significance (p_uncorr_ < 0.001) the Fantasy subscale was positively associated with right cerebellar lobule 4-5, right frontal inferior pars triangularis, left frontal inferior pars orbitalis, right cuneus, right lingual gyrus, right hippocampus, and with left precuneus. Other positive uncorrected associations (p_uncorr_ < 0.001) were found between Personal Distress subscale and volumes in left superior temporal gyrus, and Empathic Concern subscale and volumes in left frontal superior gyrus. Negative uncorrected associations (p_uncorr_ < 0.001) were found between Perspective Taking and volumes in right supplementary motor area, right precentral gyrus, right frontal superior gyrus, and right frontal inferior pars opercularis, and in left postcentral gyrus. and between Personal Distress subscale and left medial superior frontal gyrus (Supplementary Table 1).

The volumetric modifications found to be associated to empathic abilities are consistent with literature data. Namely, the volumes of inferior frontal gyrus, somatosensory cortex, precuneus, and cuneus are consistently associated with the individual differences in cognitive empathy (Avenanti et al., 2009; Keysers and Gazzola, 2006), while the volume of temporal superior gyrus is associated with the individual differences in affective empathy (Mackes et al., 2018). These findings support the evidence for cognitive and affective empathy being represented by cortical correlates.

**References**

- Avenanti, A., Minio Paluello, I., Bufalari, I., Aglioti, S., 2009. The pain of a model in the personality of an onlooker: Influence of state-reactivity and personality traits on embodied empathy for pain. *NeuroImage* 44, 275–283.

- Keysers, C., Gazzola, V., 2006. Towards a unifying neural theory of social cognition, in: *Progress in Brain Research*. Elsevier, pp. 379–401.

- Mackes, N.K., Golm, D., O’Daly, O.G., Sarkar, S., Sonuga-Barke, E.J.S., Fairchild, G., Mehta, M.A., 2018. Tracking emotions in the brain – Revisiting the Empathic Accuracy Task*. NeuroImage* 178, 677–686

**Table 1S| Regional gray matter Volumes (ROI-based analyses) and IRI subscales**

| **Label for peak** | **Side** | **Extent (n voxels)** | **t** | ***p(uncorr)*** | | **equivZ** | **x,y,z (mm)** |  |  |  |
| --- | --- | --- | --- | --- | --- | --- | --- | --- | --- | --- |
| **Fantasy** |  |  |  | |  |  |  |  |  |  |
| Precuneus | L | 69 | 4.08 | | < 0.001 | 3.84 | -10, -57, 24 |  |  |  |
| **Perspective Taking** |  |  |  | |  |  |  |  |  |  |
| Supplementary Motor Area | R | 116 | 3.90 | | < 0.001 | 3.68 | 3, -12, 52 |  |  |  |
| Supplementary Motor Area | R | 96 | 3.71 | | < 0.001 | 3.52 | 14, -18, 67 |  |  |  |
| Somatosensory Cortex | L | 257 | 4.11 | | < 0.001 | 3.86 | -52, -12, 31 |  |  |  |
| **Empathic Concern** |  |  |  | |  |  |  |  |  |  |
| Somatosensory Cortex | L | 56 | 3.37 | | < 0.001 | 3.23 | -26, -34, 79 |  |  |  |
| **Personal Distress** |  |  |  | |  |  |  |  |  |  |
| Somatosensory Cortex | R | 94 | 3.99 | | < 0.001 | 3.76 | 58, -21, 48 |  |  |  |
| Somatosensory Cortex | L | 86 | 3.81 | | < 0.001 | 3.61 | -36, -13, 42 |  |  |  |

Abbreviations:

P = significance (uncorrected) at the peak level

L = left

R = right

Coordinates are in Montreal Neurological Institute (MNI) space.

**Table 2S| Regional gray matter Volumes (Voxel Based Morphometry) and IRI subscales**

| **Label for peak** | **Side** | **Extent (n voxels)** | **t** | ***p(uncorr)*** | **equivZ** | **x,y,z (mm)** |  |  |  |
| --- | --- | --- | --- | --- | --- | --- | --- | --- | --- |
| **Fantasy** |  |  |  |  |  |  |  |  |  |
| Cuneus | R | 274 | 4.37 | < 0.001 | 4.08 | 15, -78, 25 |  |  |  |
| Inferior Frontal Gyrus, Pars Triangularis | R | 54 | 4.24 | < 0.001 | 3.97 | 42, 33, 3 |  |  |  |
| Precuneus | L | 58 | 3.93 | < 0.001 | 3.84 | -10, -57, 24 |  |  |  |
| Lingual Gyrus | R | 70 | 3.93 | < 0.001 | 3.71 | 21, -66, -2 |  |  |  |
| Hippocampus | R | 98 | 3.79 | < 0.001 | 3.59 | 34, -9, -14 |  |  |  |
| Inferior Frontal Gyrus, Pars Orbitalis | L | 89 | 3.72 | < 0.001 | 3.53 | -46, 38, -8 |  |  |  |
| Cerebellum lobule 4-5 | R | 58 | 3.60 | < 0.001 | 3.43 | 16, -43, -9 |  |  |  |
| **Perspective Taking** |  |  |  |  |  |  |  |  |  |
| Postcentral gyrus | L | 308 | 4.11 | < 0.001 | 3.86 | -52, -12, 31 |  |  |  |
| Supplementary Motor Area | R | 116 | 3.90 | < 0.001 | 3.68 | 3, -12, 52 |  |  |  |
| Precentral Gyrus | R | 128 | 3.80 | < 0.001 | 3.60 | 22, -18, 67 |  |  |  |
| Superior Frontal Gyrus | R | 75 | 3.69 | < 0.001 | 3.51 | 20, 0, 55 |  |  |  |
| Inferior Frontal Gyrus, pars opercularis | R | 67 | 3.48 | < 0.001 | 3.33 | 46, 9, 10 |  |  |  |
| **Personal Distress** |  |  |  |  |  |  |  |  |  |
| Superior Temporal Gyrus | L | 172 | 4.21 | < 0.001 | 3.94 | -57, -31, 12 |  |  |  |
| Medial Superior Frontal Gyrus | L | 401 | 3.94 | < 0.001 | 3.71 | -2, 27, 45 |  |  |  |
| **Empathic Concern** |  |  |  |  |  |  |  |  |  |
| Superior Frontal Gyrus | L | 51 | 3.96 | < 0.001 | 3.74 | -27, 39, 37 |  |  |  |

Abbreviations:

*P* = significance (uncorrected) at the peak level

L = left

R =right

Coordinates are in Montreal Neurological Institute (MNI) space.

**
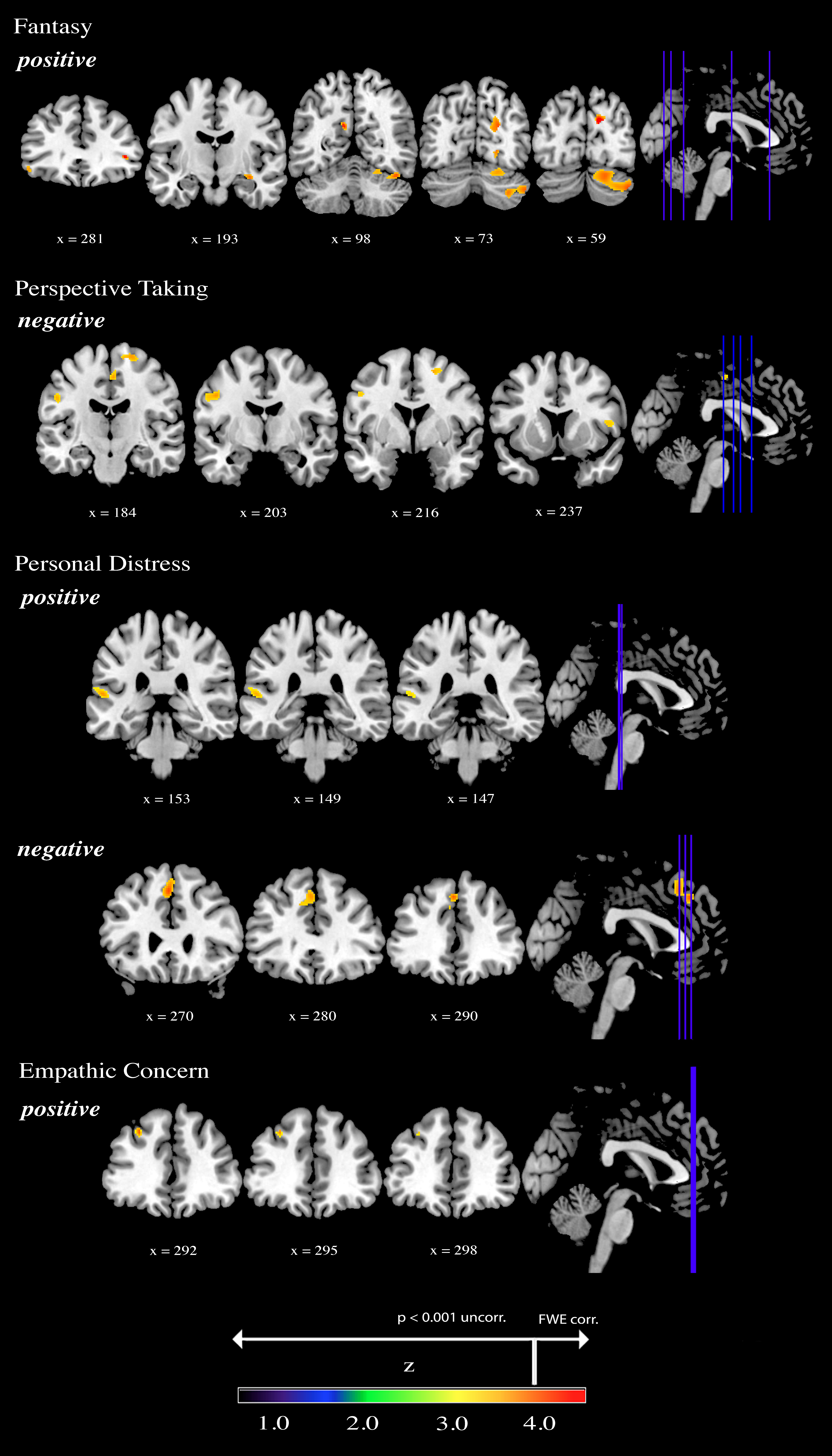
FIGURE 1S | Association between whole-brain grey matter volumes and IRI subscales.**

**FIGURE 1S. Associations between whole-brain gray matter volumes and IRI subscales.** The figure represents the negative and positive associations between IRI subscales and whole-brain gray matter volumes reported in Table 2S. Coordinates are in Montreal Neurological Institute (MNI) space. Z above colorbar indicates normalized t-values. In figure left is left.
